# Supplementary material for: Stimulus-Induced Narrowband Gamma Oscillations are Test–Retest Reliable in Human EEG
Source: Cereb Cortex Commun. 2022 Jan 7;3(1):tgab066. doi: 10.1093/texcom/tgab066 (PMC8790174; doi:10.1093/texcom/tgab066)
Supplement: Supplementary_Figures_tgab066 [file supplementary_figures_tgab066.docx]

**Supplementary Figures**

**
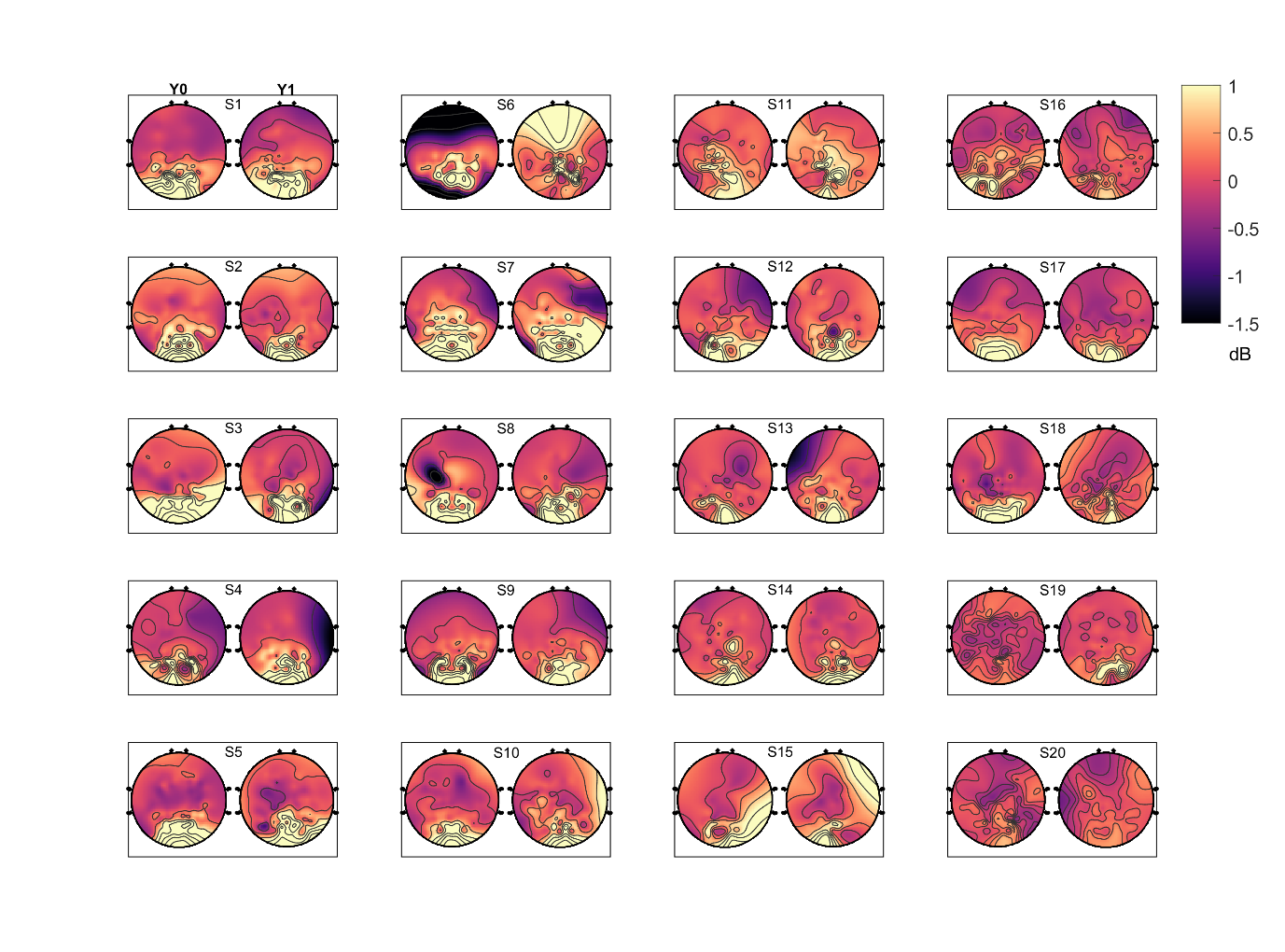
**

**Supplementary Figure S1. Scalp maps for average gamma power are also consistent between baseline and follow-up sessions.** Average of slow and fast gamma power across common good bipolar electrodes is plotted as a scalp map for 20 female subjects. Same subject order as Figure 1. The color bar on the right denotes the power ratio in dB units.


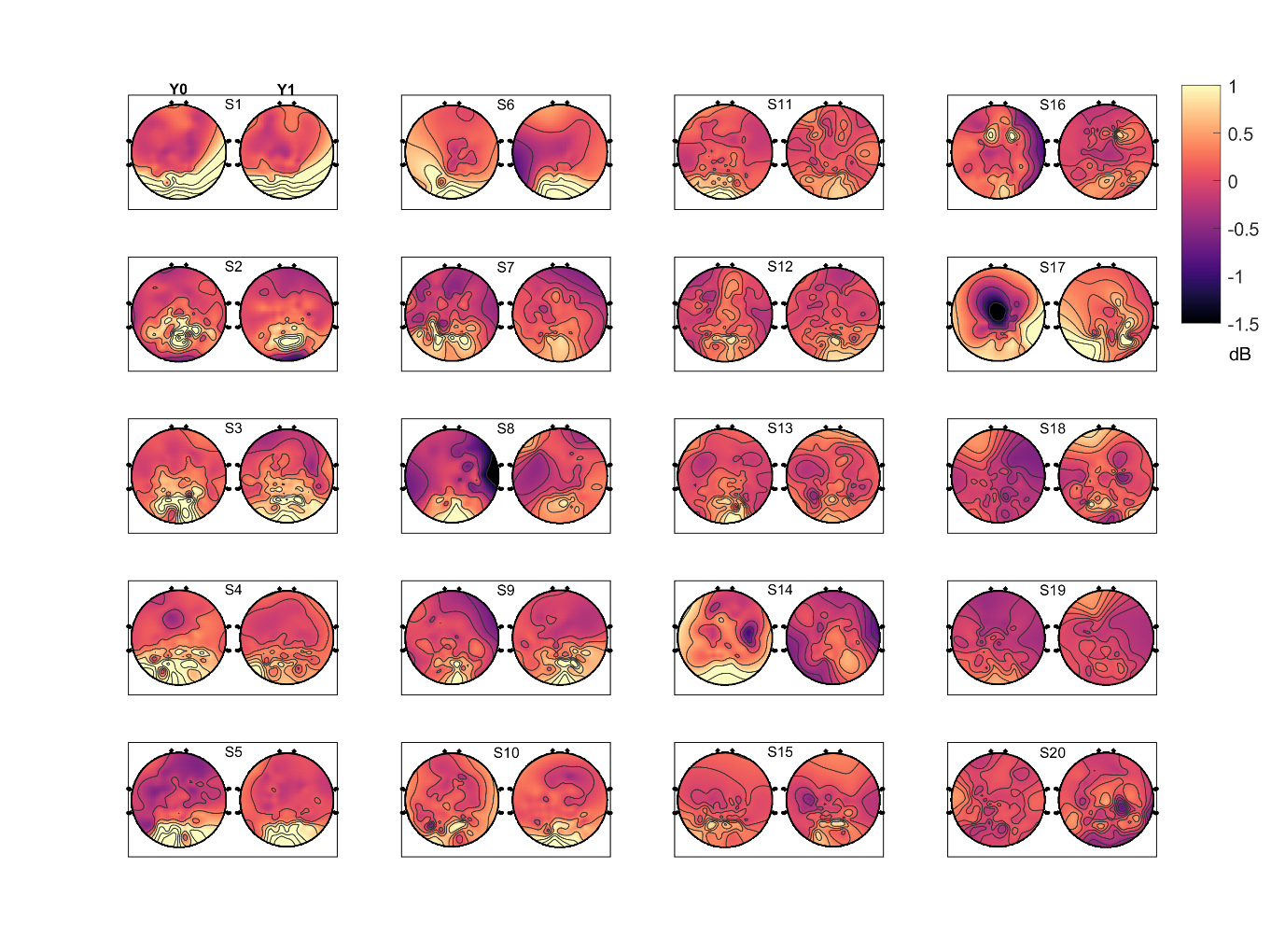


**Supplementary Figure S2.** Same as Supplementary Figure S1 for 20 male subjects.


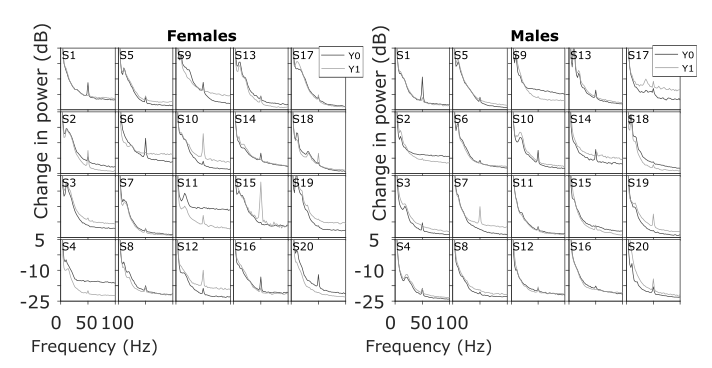


**Supplementary Figure S3.** **Resting state Power Spectral Density (PSD) profiles are also consistent across baseline and follow-up in males and females.** Same as Fig. 3 for the absolute PSDs on the resting state data during the baseline period of the experimental data.


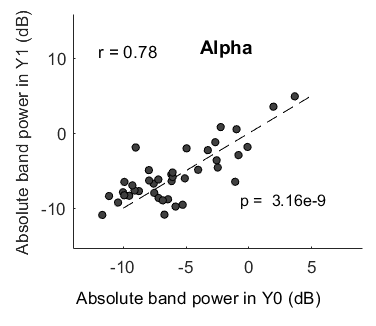


**Supplementary Figure S4. Resting state absolute alpha power is correlated across baseline and follow-up**. Same as Fig. 5 for resting state absolute alpha power (computed over the baseline period).
